# Nitrogen Deposition Enhances Carbon Sequestration by Plantations in Northern China

**DOI:** 10.1371/journal.pone.0087975

**Published:** 2014-02-03

**Authors:** Zhihong Du, Wei Wang, Wenjing Zeng, Hui Zeng

**Affiliations:** 1 Department of Ecology, College of Urban and Environmental Sciences, and Key Laboratory for Earth Surface Processes of the Ministry of Education, Peking University, Beijing, China; 2 Key Laboratory for Urban Habitat Environmental Science and Technology, Peking University Shenzhen Graduate School, Shenzhen, China; North Carolina State University, United States of America

## Abstract

Nitrogen (N) deposition and its ecological effects on forest ecosystems have received global attention. Plantations play an important role in mitigating climate change through assimilating atmospheric CO_2_. However, the mechanisms by which increasing N additions affect net ecosystem production (NEP) of plantations remain poorly understood. A field experiment was initialized in May 2009, which incorporated additions of four rates of N (control (no N addition), low-N (5 g N m^−2^ yr^−1^), medium-N (10 g N m^−2^ yr^−1^), and high-N (15 g N m^−2^ yr^−1^)) at the Saihanba Forestry Center, Hebei Province, northern China, a locality that contains the largest area of plantations in China. Net primary production (NPP), soil respiration, and its autotrophic and heterotrophic components were measured. Plant tissue carbon (C) and N concentrations (including foliage, litter, and fine roots), microbial biomass, microbial community composition, extracellular enzyme activities, and soil pH were also measured. N addition significantly increased NPP, which was associated with increased litter N concentrations. Autotrophic respiration (AR) increased but heterotrophic respiration (HR) decreased in the high N compared with the medium N plots, although the HR in high and medium N plots did not significantly differ from that in the control. The increased AR may derive from mycorrhizal respiration and rhizospheric microbial respiration, not live root respiration, because fine root biomass and N concentrations showed no significant differences. Although the HR was significantly suppressed in the high-N plots, soil microbial biomass, composition, or activity of extracellular enzymes were not significantly changed. Reduced pH with fertilization also could not explain the pattern of HR. The reduction of HR may be related to altered microbial C use efficiency. NEP was significantly enhanced by N addition, from 149 to 426.6 g C m^−2^ yr^−1^. Short-term N addition may significantly enhance the role of plantations as an important C sink.

## Introduction

Terrestrial ecosystems sequester nearly 30% of anthropogenic carbon (C) emissions, offering the most effective, yet natural, means to mitigate climate change [1]. Nitrogen (N) is a major limiting nutrient to plant growth in most terrestrial ecosystems [2] and thus affects C sequestration in terrestrial ecosystems [3]. Human activity has led to a significant increase in N deposition owing to industrialization, agricultural practices, and the combustion of fossil fuels [4]–[6]. Numerous studies have shown that N deposition can increase net ecosystem production (NEP), as an indicator of ecosystem C sequestration [7]–[9]. However, the magnitude of the increased NEP following N addition varied greatly from 24.5 to 225 kg C per kg N [10]–[12]. Therefore, there is an urgent need to explore the mechanisms underlying this effect.

NEP is determined by the difference between net primary production (NPP) and soil heterotrophic respiration (HR). One important issue that needs addressing is how additional N affects the process of plant growth and thus enhances NPP. Many studies have attributed the increased tree growth to significantly higher foliar N concentrations in fertilized plots [8], [13]–[15]. The increased foliar N concentrations could improve biomass production through the following three pathways: by increasing the uptake of CO_2_ [16]–[18], by increasing water-use efficiency of foliage via altering CO_2_ assimilation and stomatal conductance [19], and by reducing the thermally dissipated light [20]. At the same time, N addition may also decrease leaf N resorption [21], and thus increase litter N concentrations. Consequently, more available N is released via decomposition to supply plant growth [22]. However, little research has been conducted to comprehensively analyze the mechanism of plant biomass growth caused by N addition.

How soil respiration (SR) responds to N addition is also relevant. SR consists of autotrophic respiration (AR, respiration by live roots, rhizospheric microorganism, and mycorrhizal fungi) and HR, which mainly originates from microbial decomposition of soil organic matter. With N addition, AR was either inhibited by decreasing the below-ground C allocation and fine root biomass of trees [23] or promoted by increasing the N concentration in fine roots [24]–[28]. At the same time, the enhanced tree growth caused by N addition is also likely to lead to more plant photosynthate being transported from above ground to below ground, thus increasing AR. HR is also commonly considered to be related to microbial biomass and activity [29]–[31]. For instance, decreased HR was observed along with a consistent decrease in microbial biomass and extracellular enzyme activity [32]. Soil acidification caused by N deposition [33] is also a potential factor that could contribute to decreased HR. However, the inherent reasons concerning the responses of AR and HR to N addition are poorly understood.

Although there have been numerous studies investigating the effects of N deposition on ecosystem C sequestration [27], [34], [35], most of them focused on natural forests. Plantations are becoming a key component of world forest resources and play important roles in the context of overall sustainable forest management. Well-designed, multi-purpose plantations can reduce pressure on natural forests, restore some ecological services provided by natural forests, and mitigate climate changes through direct C sequestration [36]. However, there remain great uncertainties in the potential of plantations to sequestrate C [37]. Compared with natural forests, plantations appear to have lower NPP, root biomass, and soil microbial biomass [37]. Whether plantations have the same ecosystem C sequestration capacity as natural forests remains to be confirmed [38]–[40]. Among a few studies, increased ecosystem C sequestration with N deposition has been observed [41], [42]. However the underlying mechanisms by which N increases the plant C accumulation and affects SR and its autotrophic and heterotrophic components are still poorly understood.

In China, the total plantation area reached 5.33×10^7^ ha in 1998, accounting for 30% of the total forest area of China and 29% of the world's total plantation area [43]. C accumulation in China is mainly ascribable to its extensive afforestation efforts, as 80% of the observed increase in tree C stocks in China occurred on its 213,106 ha of plantations [43]. These reforestation and afforestation programs are considered to influence C storage in China. Thus, to assess the C sequestration capacity of plantations and optimize their role as C sinks, it is necessary to systematically explore ways in which N deposition affects C sequestration. Consequently, a 3-year field N addition experiment was conducted in the Saihanba Forestry Center, Hebei Province, northern China, which contains the largest area of plantations in China, with the dominant species being *Pinus sylvestris* var. *mongolica* (Mongolia pine). NPP, SR, and its autotrophic and heterotrophic components were measured. Relevant influential factors were also measured, including plant tissue C and N concentrations (foliage, litter, and fine roots), microbial biomass C, microbial community composition, potential extracellular enzyme activities (EEAs), and soil pH values. The study aimed to address three questions: (1) how does the N addition affect NPP? (2) What are the responses of SR and its autotrophic and heterotrophic components to N addition? (3) What is the effect of N addition on NEP? We hypothesized that: (1) N addition would increase NPP via increasing foliage or litter N concentrations; (2) AR would remain stable because of the contrasting effects from decreasing below-ground C allocation and fine root biomass and increased fine root N concentrations and photosynthate transport from above ground to below ground; HR would be reduced because of decreased microbial biomass, inhibited microbial activity, and reduced pH values; and (3) NEP would be enhanced because of the increasing NPP and decreased HR.

## Materials and Methods

### Ethics Statement

The administration of the Saihanba Forestry Center gave permission for the use of their plantation for our study site. We confirm that the field studies did not involve endangered or protected species.

### Site description

The study was conducted at the Saihanba Forestry Center in Hebei Province, northern China (117°12′–117°30′ E, 42°10′–42°50′ N, 1400 m a.s.l.). The study area belongs to a typical forest-steppe ecotone of the temperate area. The climate is semi-arid and semi-humid, with a long and cold winter (November to March), and a short spring and summer. Annual mean air temperature and precipitation over the period from 1964 to 2004 were −1.4°C and 450.1 mm, respectively. The soils are predominantly sandy. The study site is located in the largest area of plantations in China, with the dominant species being *Pinus sylvestris* var. *mongolica*. The herbaceous layer is dominated by *Carex rigescens, Thalictrum aquilegifolium*, *Galium verum*, *Geum aleppicum*, *Artemisia tanacetifolia*, and *Agrimonia pilosa*.

### Experimental treatments

The N addition experiment was initiated in May 2009. Urea solution was evenly sprayed once a month from May to September with the same dose each year. Four N addition treatments (in three replicates) were established, including a control (without N added), low N (5 g N m^−2^ yr^−1^), medium N (10 g N m^−2^ yr^−1^), and high N (15 g N m^−2^ yr^−1^). Twelve plots, each of 20 m × 20 m dimensions were established, each surrounded by a 10-m wide buffer strip. All plots and treatments were randomly laid out. During each application, the fertilizer was weighed, mixed with 10 L of water, and applied to each plot below the canopy using a backpack sprayer. The control plot received 10 L of water without N.

### Field measurements

#### Biomass production and accumulation

An allometric method was used to estimate biomass production through establishing the relationship between component biomass (foliage, branches, stem, and roots) and diameter at breast height (DBH) and tree height (H) [44]. In July 2010, stems were cut at the soil surface in the area near our experimental plots. Total tree heights, length of live crown, DBH, and diameter at the base of the live crown were measured and recorded. All foliage on each live branch was collected and weighed. All live and dead branches from each canopy position were cut and weighed separately. The stems were cut into 1-m sections and weighed. Litter from each deforested tree was carefully collected and weighed. The entire root system of the sample trees was excavated using a combination of a pulley device and manual digging, and cleaned of adhering soil. The fresh mass of each component was determined to the nearest 1 g using an electronic balance. All of these procedures were conducted in the field immediately after the tree was felled. The total biomass was calculated as the sum of foliage, branch, litter, stem, and root biomass.

An allometric equation was established as: 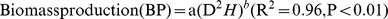
where H is the height of trees (m), D is DBH (cm), and *a* and *b* are regression constants (*b* = 0.70, *a* = 107.01). DBH was recorded on all living stems in each plot in July 2010 and July 2012. The height of each living tree was measured using a DME (Haglöf Vertex IV, Sweden). Because biomass production (BP) constitutes the largest fraction of NPP, BP is commonly used as a proxy for NPP [45]–[48]. It is important to note that NPP includes numerous C-consuming processes such as plant growth, root exudation, and C allocation to symbionts [49]. The NPP in our study was thus underestimated. We used 50% as the C concentration in plant tissue [28]. The net primary production was calculated by the following equation:
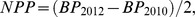
where *NPP* is the net primary productivity (g C m^−2^ yr^−1^) and *BP* is the estimated biomass production (g C m^−2^ yr^−1^) in 2012 and 2010. The annual net ecosystem production (NEP, g C m^−1^ yr^−1^) was calculated as the annual NPP minus annual soil HR.

#### Fine root biomass

In July 2011 and 2012, five soil core samples were taken randomly using a 5.8-cm-diameter soil corer around the trees in each replicate plot, causing as little disturbance as possible to the surrounding soil. The roots were transported to the laboratory where they were washed free of soil, dried at 70°C, and weighed.

#### SR and its autotrophic and heterotrophic components

SR was measured using a Li-8100 soil CO_2_ flux system (LI-COR Inc. Lincoln, NE, USA). Measurements were conducted at least once per month from May to October in 2011 and 2012. There were five subsamples (i.e., SR collars) in each plot. We used two kinds of soil collars in each plot to measure total SR and HR. A shallow surface collar (10 cm inside diameter, 6 cm height) that penetrated 3 cm into the soil was used to measure SR. The other kind of collar (10 cm inside diameter, 35 cm height), which was used for HR measurement, was inserted 30 cm into the soil with a 5-cm height above the soil surface. Because the majority of roots are found within the upper 30 cm of the soil profile in this forest (data not shown), these deeper collars should eliminate the majority of live roots and their contributions to respiration. All the polyvinyl chloride (PVC) collars were installed 6 months prior to the first measurements to minimize any disturbance of the soil environment. SR in the growing season was obtained from the monthly data directly measured in the field experiment using linear extrapolation methods. Winter SR was obtained from the data of Wang et al. (2010) [50] from the same study site.

### Laboratory analyses

#### Plant chemical analyses

Five subsamples were collected in each plot for chemical analyses. Green foliage was sampled from vigorously growing trees in late July 2012 using a pole pruner and a steel ladder. Foliar litter was collected from litter traps. Fine root samples were selected after the soil was passed through a 2-mm sieve. All the green foliage, foliar litters, and fine roots were dried at 60°C to constant mass, and ground using an intermediate mill (0.5-mm mesh screen) to generate homogeneous samples for chemical analysis. The C and N concentrations were measured using an element analyzer (Vario EL III, Elementar, Hanau, Germany).

#### Soil chemical analyses

In July 2012, mineral soils were sampled at 0–10 cm depth from five random locations per plot using 5.8-cm-diameter soil corers. Once collected, the soils were immediately placed in a cooler and transported to the nearby laboratory (less than 30 min travel time per site). The cores from each plot were then combined and frozen for later processing. Within 24 h, frozen soils were allowed to thaw at room temperature. Plant litter in the upper layer, as well as all the coarse and fine roots, was carefully removed. The soils were then separated into four sub-samples for laboratory analysis, including pH, microbial biomass C and N, microbial community composition (PLFAs), and potential EEAs.

Air-dried soil had any roots removed, and was passed through a 2-mm sieve. Soil pH was determined using a 1∶5 soil:water ratio with a pH meter (Model PHS-2; INESA Instrument, Shanghai, China).

MBC and MBN were measured using the chloroform fumigation extraction technique [51], [52]. Two replicate samples, one unfumigated and one fumigated with alcohol-free CHCl_3_ for 24 h, were pre-incubated at 25°C for 7 days and then extracted with 0.5 mol/L K_2_SO_4_ (1∶2.5 w/v). The extracts were analyzed for total dissolved C and N using a total C analyzer (TOC-500; Shimadzu, Kyoto, Japan). The microbial biomass was calculated as the difference in extractable C and N between the fumigated and unfumigated soils. The efficiency factors used to calculate the respective MBC and MBN were *K_C_* = 0.379 [52] and *K_N_* = 0.54 [51].

Phospholipid fatty acids (PLFAs) analysis was used to assess microbial community composition. PLFAs were extracted and analyzed using a procedure described by [53]. Briefly, the soil was extracted in a single-phase mixture of chloroform: methanol: citrate buffer (1: 2: 0.8) [54]. After extraction, the lipids were separated into neutral lipids, glycolipids, and polar lipids (phospholipids) on a silicic acid column. The phospholipids were methylated and separated on a gas chromatograph equipped with a flame ionization detector. Peak areas were quantified by adding methyl nonadecanoate fatty acid (19:0) as the internal standard before the methylation step. Peaks were identified by chromatographic retention time and a standard qualitative mix in the range of C9–C30 using a microbial identification system (Microbial ID Inc., Newark, DE, USA). The fatty acid 18: 2ω6, 9 was recognized as the fungal biomarker [55]. The sum of the following PLFAs was used a measure of the bacterial biomass: i14:0, i15:0, a15:0, 15:0, i16:0, 10Me16:0, i17:0, a17:0, cy17:0, 17:0, br18, 10Me17:0, 18:1ω7, 10Me18:0, and cy19:0 [56].

Seven EEAs were measured, including five enzymes involving C metabolism (α-glucosidase (AG), β-1,4-glucosidase (BG), leucine aminopeptidase (LAP), β-D-cellobiosidase (CB), and xylosidase (XS)), one involving N metabolism (N-acetyl-glucosaminidase (NAG), and one involving phosphorus (P) metabolism (acid phosphatase (AP)). The measurements were conducted following the method of Saiya-Cork et al. (2002) [57]. Briefly, sample suspensions were prepared by adding 2 g of fresh soil to 90 ml of 50 mmol/L, pH 6.0 acetate buffer and homogenizing for 1 min. Continuously, 200-µl suspensions were combined with the corresponding substrate in a 96-well microplate. There were six replicate wells per sample per assay. The micro-plates were incubated at 25°C for up to 3 h. Fluorescence was then measured using a microplate reader with 365-nm excitation and 450-nm emission filters (Tecan Infinite M200, Salzburg, Austria). Finally, the concentration was divided by incubation time and dry weight soil to estimate potential enzyme activity.

### Statistical analysis

All statistical analyses were performed using SPSS statistical software (SPSS 18.0 for Windows; SPSS Inc., Chicago, IL, USA). One-way analysis of variance with Duncan's test was used to test the differences among the different N addition treatments in NPP, SR, and its AR and HR components, and in NEP, as well as plant and soil chemical parameters. Significant effects were determined at *P*<0.05 unless otherwise stated. Data was expressed as mean values ± S.E. (standard error).

## Results

### Biomass production and accumulation

**Figure 1:**
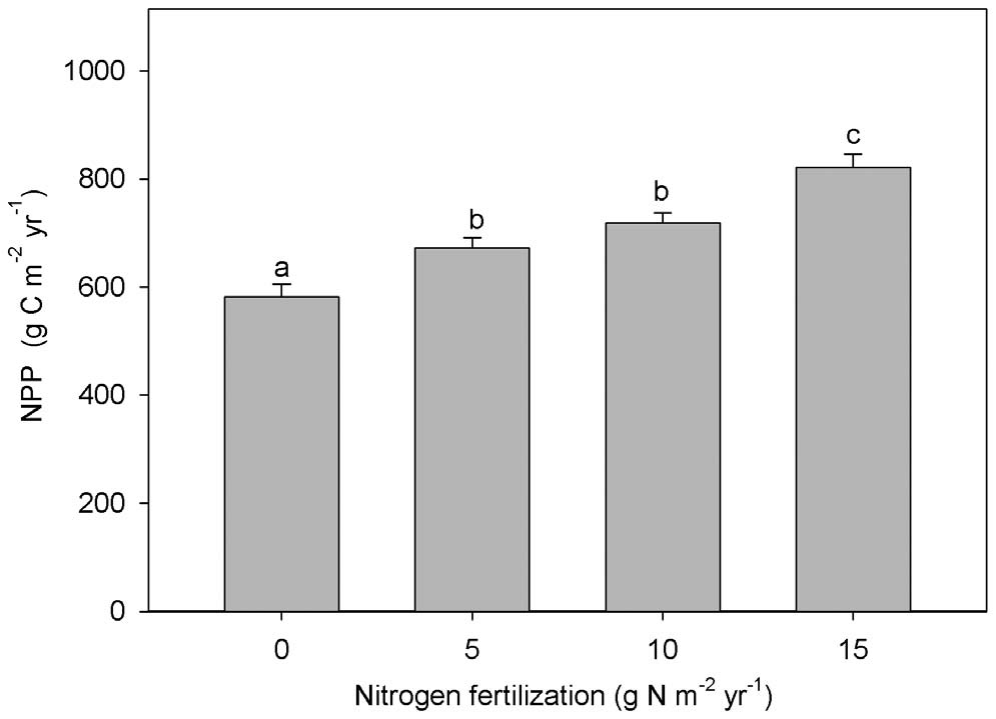
Average net primary productivity (NPP) in control and nitrogen (N) fertilized treatments. Significant differences among N treatments are indicated by different letters.

No significant differences were observed in DBH for both 2010 and 2012 among the different N addition treatments (Fig. S1a). However, in 2012, the tree height significantly increased with fertilization (Fig. S1b). The averaged NPP was 582.3±22.8 g C m^−2^ yr^−1^ in the control plots. N addition increased NPP by 15.45%, 23.51%, and 41.21%, respectively, in the low-, medium-, and high-N plots (Fig. 1). Fine root biomass showed a decreasing trend with fertilization although no significant differences were observed (Fig. S2).

### SR and its autotrophic and heterotrophic components

**Figure 2:**
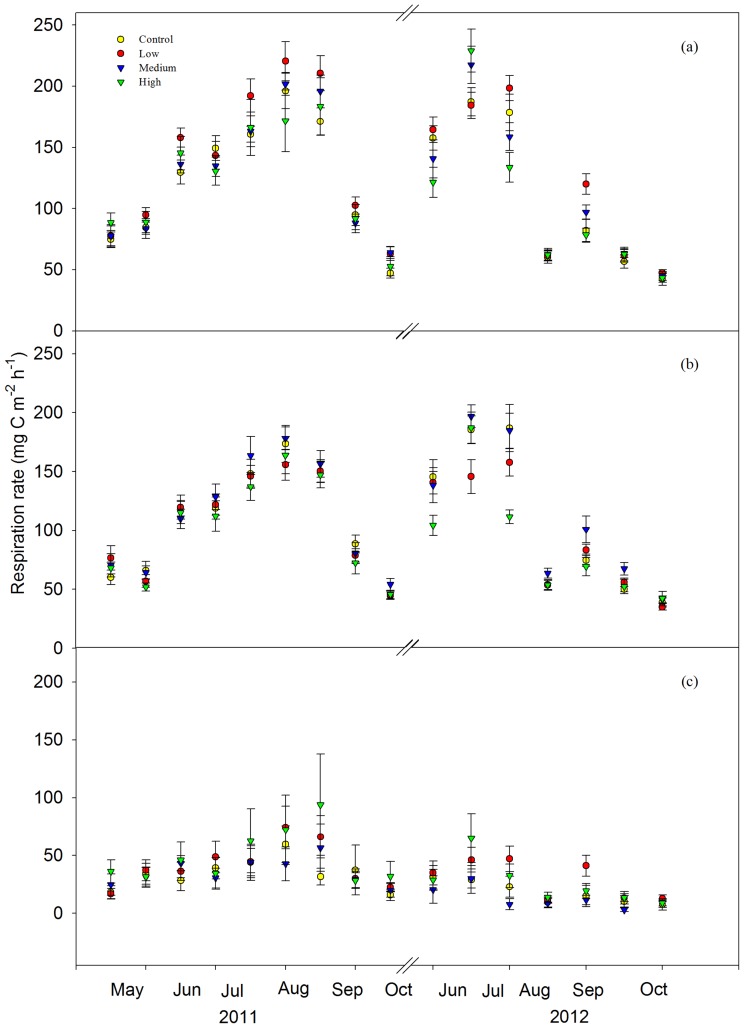
Soil respiration (a), heterotrophic respiration (b), and autotrophic respiration (c) in control (yellow circle), low-nitrogen (N) (red circle), medium-N (blue triangle), and high-N (green triangle) plots during the growing seasons of 2011 and 2012.

**Figure 3:**
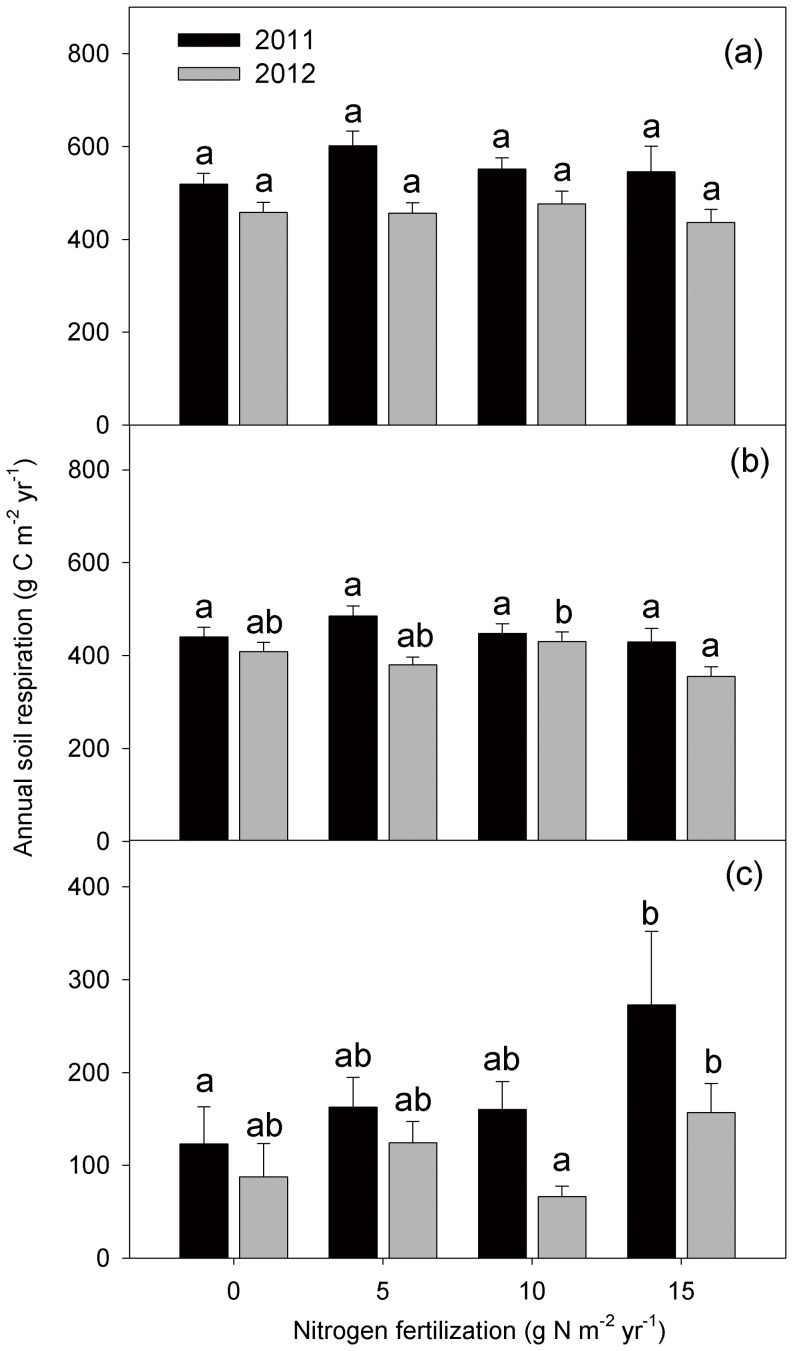
Comparison of annual soil respiration (a), autotrophic respiration (b), and heterotrophic respiration (c) among control, low-, medium-, and high-nitrogen (N) plots in 2011 (black) and 2012 (gray). Significant differences among N treatments are indicated by different letters.

Both total SR and HR followed a clear seasonal pattern with the highest rates in June–August and the lowest rates in spring and autumn for all the treatments (Fig. 2). SR was not significantly different among control and fertilized treatment plots in both 2011 (*P* = 0.43) and 2012 (*P* = 0.36) (Fig. 3a). There was no significant variance between the control and low-N treatment for AR and HR in both 2011 and 2012 (*P*>0.05) (Fig. 3b, Fig. 3c). With the fertilization gradient increasing, significantly higher AR (Fig. 3c) and lower HR (Fig. 3b) occurred in the high-N plots compared with the medium-N treatment in 2012.

### Net ecosystem productivity (NEP)

**Figure 4:**
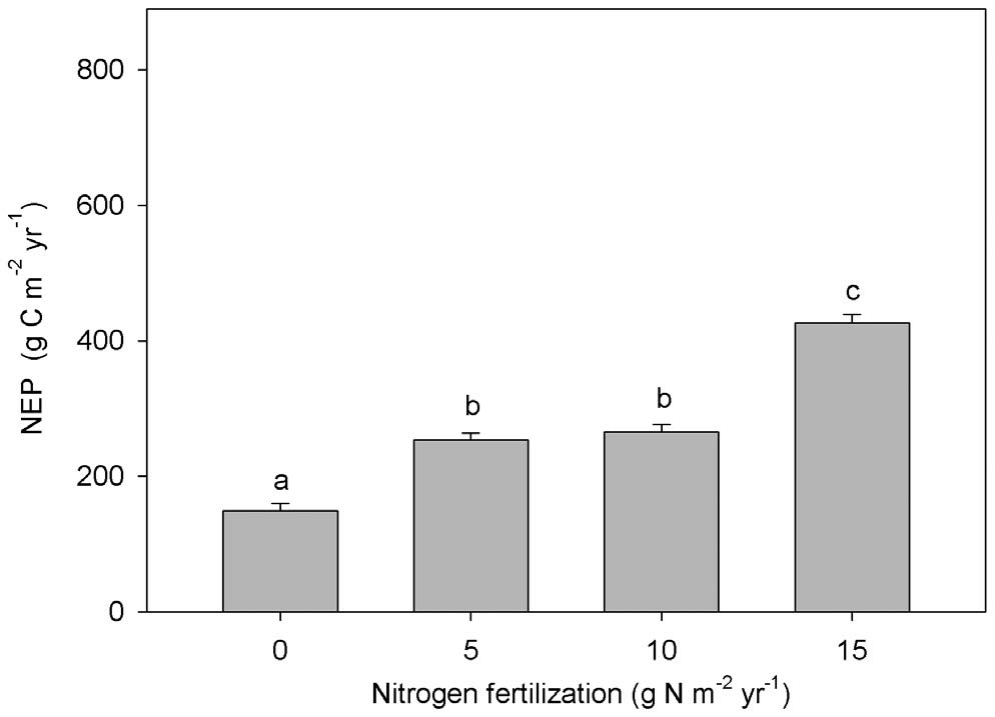
Net ecosystem productivity (NEP) in control and nitrogen (N) fertilized treatments. Significant differences among N treatments are indicated by different letters.

With a decrease in HR and increased NPP, NEP significantly increased with fertilization, from 149 to 426.6 g C m^−2^ yr^−1^ (Fig. 4). The amount of C (kg) fixed by 1 kg N/ha N addition was in the range of 116.6–209.8 kg C per kg N/ha.

### Plant chemical parameters

**Figure 5:**
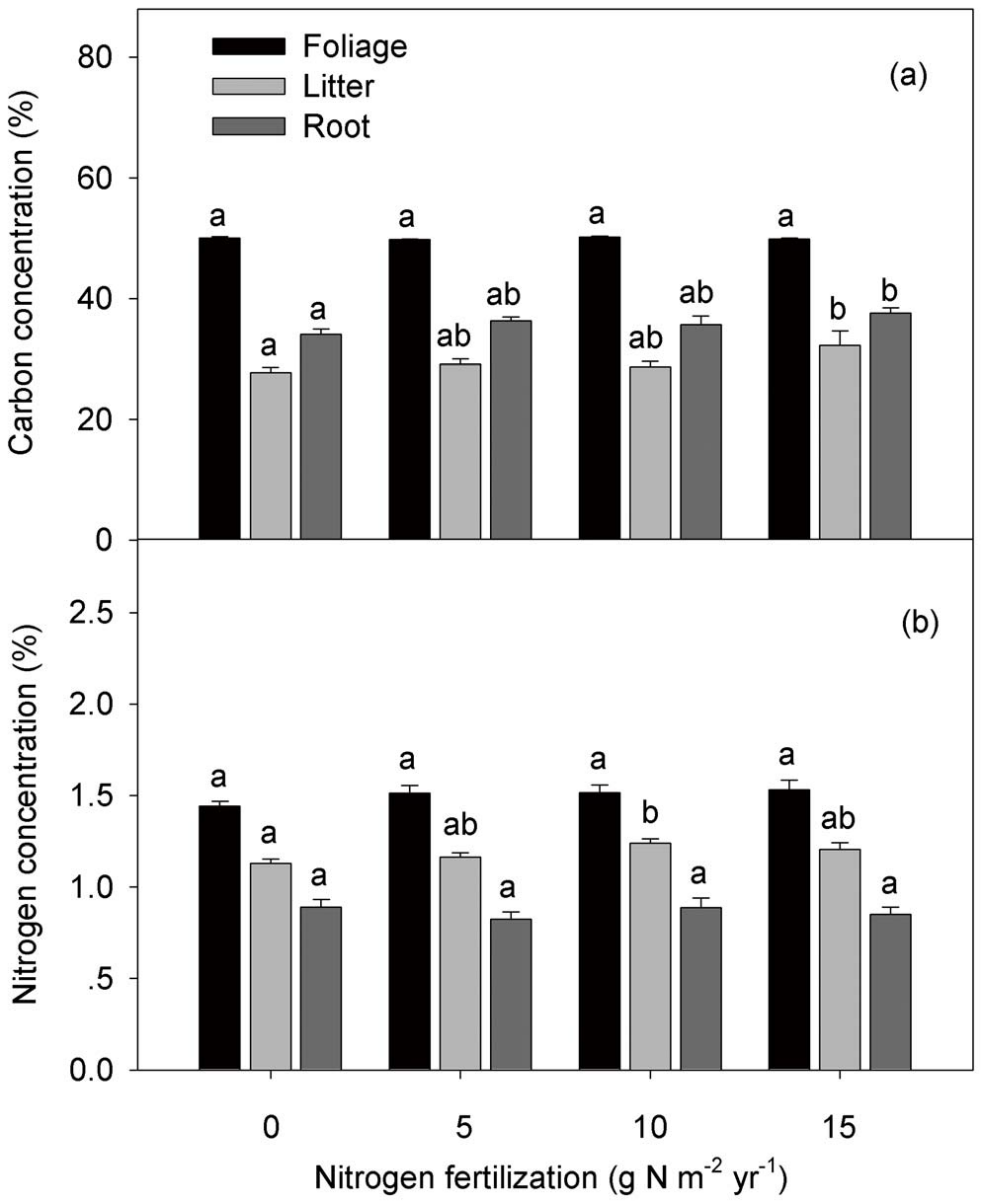
Carbon (a) and nitrogen (b) concentrations of foliage, litter, and fine roots in control and nitrogen (N) fertilized treatments. Significant differences among N treatments are indicated by different letters.

N addition significantly increased foliar N concentrations in the herbaceous layer (Fig. S3). However, N concentrations of the tree foliage showed no significant differences among the control and fertilization treatments (Fig. 5). C and N concentrations of foliar litter were significantly higher in the high-N and medium-N plots than in the control (Fig. 5). Fine root N concentrations showed no significant differences while fine root C concentrations increased by 10.28% in the high-N plots compared with the control (Fig. 5).

### Soil chemical parameters

**Figure 6:**
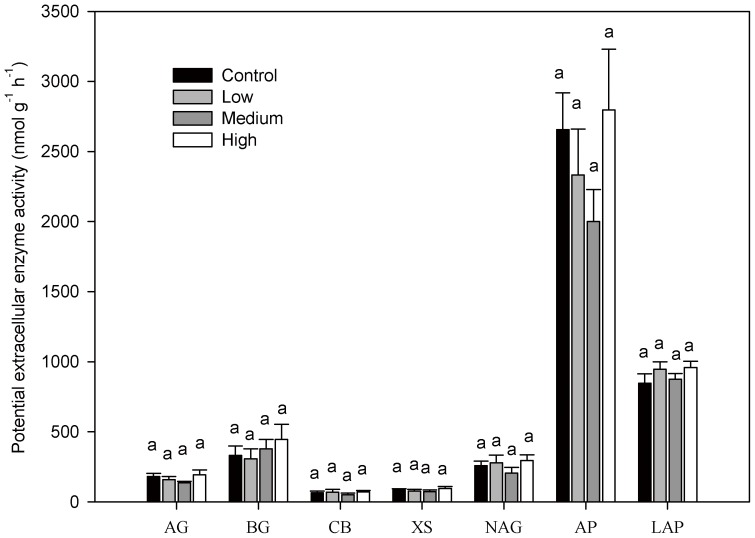
Potential extracellular enzyme activity at a soil depth of 0–10 cm in control and fertilized plots measured in 2012. Significant differences among nitrogen (N) treatments are indicated by different letters. AG  =  α-glucosidase; BG  =  β-1,4-glucosidase; CB  =  β-D-cellobiosidase; XS  =  xylosidase; NAG  =  N-acetyl-glucosaminidase; AP  =  acid phosphatase; LAP  =  leucine aminopeptidase.

Soil pH significantly decreased with fertilization (Fig. S4). MBC and MBN were 132.67–145.68 mg C kg^−1^ dry soil and 48.98–60.59 mg C kg^−1^ dry soil, respectively. Although there were no significant differences among the control and fertilized treatments, MBC and MBN generally increased along the fertilization gradient (Table 1). Neither bacterial nor fungal biomass varied significantly with N addition (Table 1). The activities of all seven enzymes involving C, N, and P metabolism showed no significant differences between the control and fertilized plots (Fig. 6).

**Table 1:** Effects of nitrogen (N) addition on microbial biomass carbon (MBC), microbial biomass N (MBN), bacterial biomass (BB), and fungal biomass (FB).

Microbial properties	N treatment			
	Control	Low N	Medium N	High N
MBC	132.67±22.39^a^	157.55±18.31^a^	162.14±22.51^a^	145.68±21.05^a^
MBN	48.9±82.46^a^	51.88±4.81^a^	55.55±66.62^a^	60.59±5.98^a^
BB	17.0±1.52^a^	18.94±2.73^a^	16.19±2.53^a^	12.91±1.58^a^
FB	7.08±0.75^a^	9.62±2.15^a^	7.41±1.21^a^	6.19±0.11^a^

Data are expressed as mean ± S.E. (standard error). Different superscript letters indicated significant differences among N treatment plots (*P*<0.05).

## Discussion

### Effects of N addition on NPP

N fertilization significantly increased NPP by 15.4–41.2% (Fig. 1) through the vertical growth of trees (Fig. S1). This maximum rate of increase of NPP is more than twice the average level of temperate forests (19.5%) [58]. The increased NPP could not be related to fresh foliar N concentrations, because no significant differences occurred between the control and fertilized plots (Fig. 5). This is inconsistent with commonly observed increases in foliar N with N addition [59], [13]–[15]. For instance, May et al. (2005) [15] found that foliar N concentrations averaged 11% higher in fertilization treatments than in the control in a mixed-deciduous forest. In this study, litter N concentrations significantly increased in the fertilized plots relative to the control (*P*<0.05) (Fig. 5b), suggesting a likely decrease in leaf N resorption by fertilization [15], [60], [61]. Litter with higher N concentration would be easily decomposed by microbes and release large amounts of available N for plant growth, thus potentially increasing forest productivity [62]–[65].

Foliage N concentrations of trees showed no significant differences among control and fertilized plots (Fig. 5), while foliage N concentrations in plants in the herbaceous layer significantly increased (Fig. S3). This may be because of differences in leaf shape. Compared with coniferous trees, the broad-leaved herbaceous plants may invest more N in foliage to produce enzymes and proteins associated with photosynthetic processes or increase their foliage area to improve photosynthesis. Increased foliage N concentrations following N addition have been commonly observed in previous studies of broadleaf species (i.e., *Acer rubrum, Liriodendron tulipifera, Prunus serotina*, *Acer saccharum*, and *Betula alleghaniensis*) [13]–[15]. Therefore, the nutrient use strategy of plants may be closely related to their foliage shape. Thus, foliage shape should be taken into consideration in the future when it comes to assessing the response of ecosystem C sequestration to N deposition.

### SR and its autotrophic and heterotrophic components

No significant differences were observed in total SR among the control and fertilized treatments (Fig. 3a), which is inconsistent with a commonly reported reduction in SR following N addition [9], [66]–[68]. Significantly higher AR in high-N plots than in the medium-N treatment was observed, although neither treatment showed any significant differences to the control (Fig. 3c). No significant differences in fine root biomass (Fig. S2) and N concentrations were observed among the different treatments (Fig. 5b), implying that live root respiration may not change with fertilization. Instead, fine root C concentrations significantly increased by 4.89%, 6.72%, and 10.28%, respectively, in the low-, medium-, and high-N addition foliage plots compared with the control treatment (Fig. 5a). This suggests an increased supply of photosynthetic products from above ground to below ground following N addition. Consequently, plant C may prime the growth and activity of mycorrhizal fungi [29] and rhizospheric microbes [23], [69]–[72], thus promoting AR. However, fertilization may also suppress rhizospheric microbial respiration to a greater extent than that in the bulk soil because of the decreased C allocation to root symbionts and exudation [68]. Therefore, there is a need to distinguish different components of AR in the future to accurately explore the internal mechanism underlying the increased AR.

HR in the high-N treatment was significantly lower than that in the medium-N plot after 3 years' fertilization, although it did not significantly differ from the control and low-N plots (Fig. 3c). Decreased HR is believed to be mainly driven by a decreased microbial biomass [29], [30] and depressed phenol oxidase activity (a lignin-degrading enzyme) [31], [73]. In contrast to most previous studies, we did not observe significant variation in the soil microbial biomass (Table 1), extracellular enzyme activity (Fig. 6), and microbial community composition (Table 1). Although soil pH was significantly lower in the high-N plots than in the control plots, it did not significantly differ from values in the medium- and low-N plots (Fig. S4). Hence, the decreased HR in the high-N plot could not be attributable to changes in the microbial biomass, extracellular enzyme activity, microbial community composition, or soil pH. The variation in HR may depend on the C-use efficiency of the decomposers (defined as the ratio of C employed in the new biomass relative to C consumed for respiration) [74]. When N availability is high, microbes may increase their efficiency leading to an efficient increase in biomass and a relatively low release of C to the atmosphere [75]. Because microbial biomass was measured only in July 2012, a greater frequency of measurement of microbial biomass should be conducted to better explore the reasons for the decrease in HR.

### Effect of N fertilization on NEP

With the increasing NPP and decreasing HR, NEP greatly increased from the control to the high-N addition plots (149 versus 426.6 g C m^−2^ yr^−1^). NEP in our control plot fell within the published range for boreal forests (40–180 g C m^−2^ yr^−1^) [76]. The amount of C fixed per unit of added N fertilizer was in the range of 116.6–209.8 kg C/ha per kg N in this study, which is broadly similar to the range proposed by Magnani et al. (2008) (175–225 kg C per kg N) [11]. Thus, short-term N fertilization can greatly increase the NEP of plantations in northern China and enhance the role of plantations as an important C sink.

However, N fertilization may also induce alterations in the availability of other nutrients such as P, potassium, and calcium, because of the intrinsic stoichemical constraints of plant growth. This could have an important influence on NEP. N deposition is also likely to be accompanied by other environmental changes including rising atmospheric CO_2_ concentrations, global warming, and soil acidification and these changes will interact with N availability in complex ways. The complexity of these interacting controls (i.e., temperature and nutrient availability) further restricts our ability to forecast future C sequestration capacities. In addition, we should be careful when extrapolating our results and mechanisms to systems with long-term N inputs. Finally, the stand age of plantations may also be a potentially influential factor in evaluating their sequestration capacity. This suggests a need for future studies in stands of various ages and incorporating long-term multi-factorial experiments.

## Conclusion

**Figure 7:**
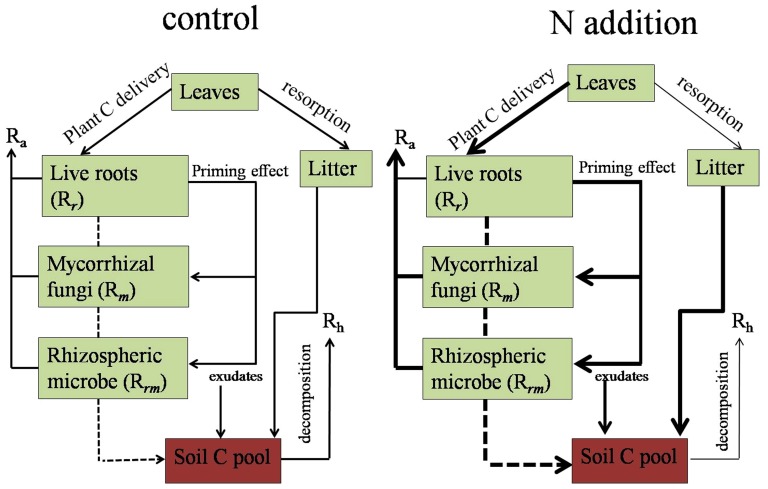
Carbon sequestration and its response to nitrogen (N) addition in plantations. R_a_ and R_h_ are autotrophic and heterotrophic respiration, respectively; R_r_ is live root respiration, R_m_ is respiration of mycorrhizal fungi, and R_rm_ is rhizospheric microbial respiration. Thick arrows represent the enhanced process and thin arrows represent the declined progress in the N addition treatment compared with the control.

This study has comprehensively analyzed the effects of N addition on biomass accumulation, SR, and its autotrophic and heterotrophic components in plantations of northern China. N addition might alter C sequestration capacity through the following possible pathways (Fig. 7): (1) increased litter N concentration because of decreased N resorption by foliage; (2) enhancement of the amount of photosynthetic products transported downward; (3) increased AR through the priming effect of plant C on rhizospheric microbial and mycorrhizal fungi activity; and (4) suppressed HR through increased microbial C use efficiency. Increasing N deposition is likely to stimulate NEP and slow the accumulation of atmospheric CO_2_. In the context of global atmospheric N deposition, we highlighted that plantations might offer an important role to mitigate the future climate change.

## Supporting Information

Figure S1 **Diameter at breast height (DBH) and height of trees with nitrogen (N) fertilization in 2010 (black) and 2012 (gray).**
(TIF)

Figure S2 **Fine root biomass among different nitrogen (N) fertilization gradients in 2012.** Significant differences among N treatments are indicated by different letters.
(TIF)

Figure S3 **Effects of nitrogen (N) addition on foliar N concentrations of herbaceous layer plants.** Significant differences among N treatments are indicated by different letters.
(TIF)

Figure S4 **Soil pH in control and nitrogen (N) treatments plots after 3 years fertilization.** Significant differences among N treatments are indicated by different letters.
(TIF)
