# Supplementary figures and images for: Nitrogen Deposition Enhances Carbon Sequestration by Plantations in Northern China
Source: PLoS One. 2014 Feb 3;9(2):e87975. doi: 10.1371/journal.pone.0087975 (PMC3912177; doi:10.1371/journal.pone.0087975)

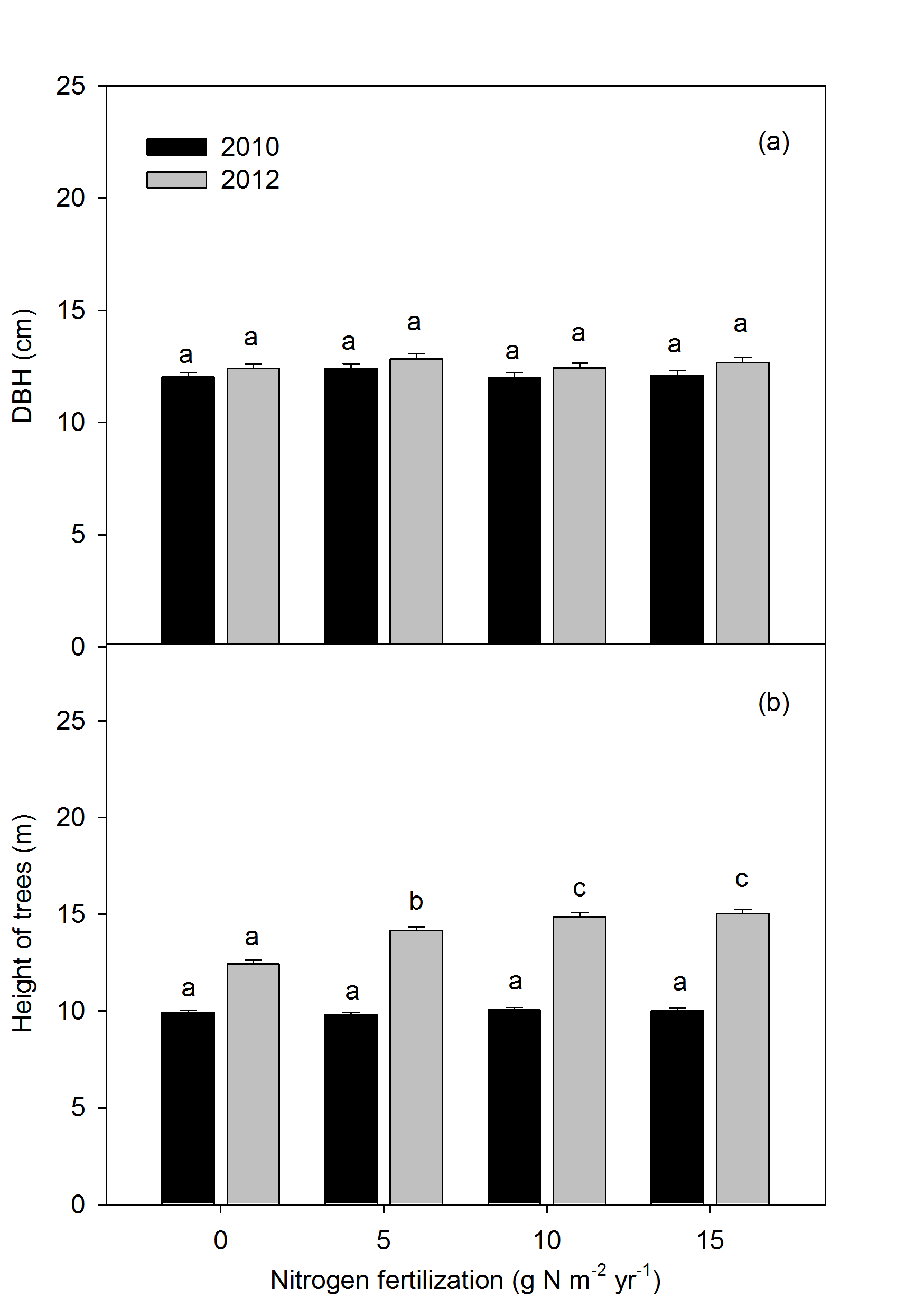

Supplement: Figure S1 — Diameter at breast height (DBH) and height of trees with nitrogen (N) fertilization in 2010 (black) and 2012 (gray). (TIF) [file pone.0087975.s001.tif]

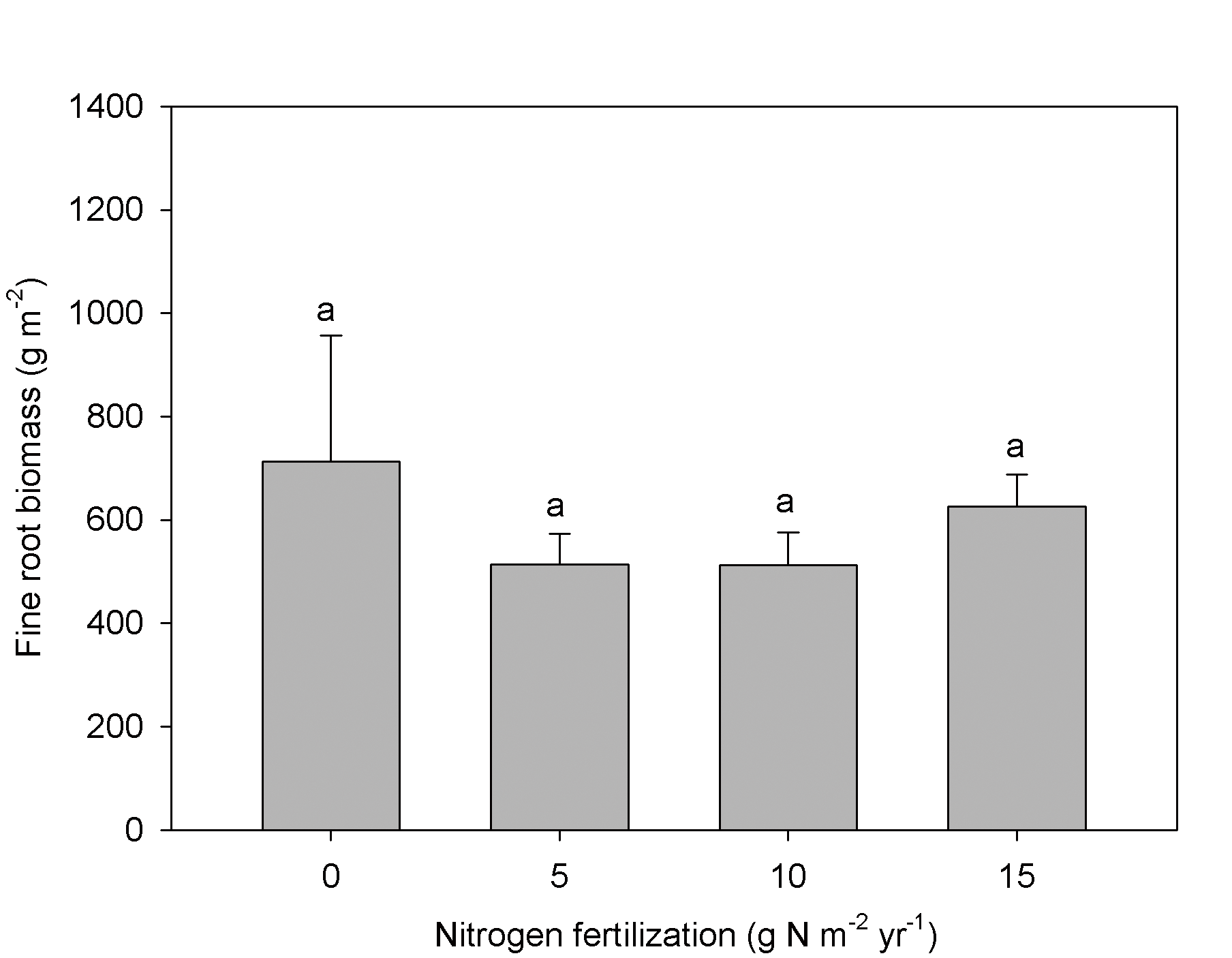

Supplement: Figure S2 — Fine root biomass among different nitrogen (N) fertilization gradients in 2012. Significant differences among N treatments are indicated by different letters. (TIF) [file pone.0087975.s002.tif]

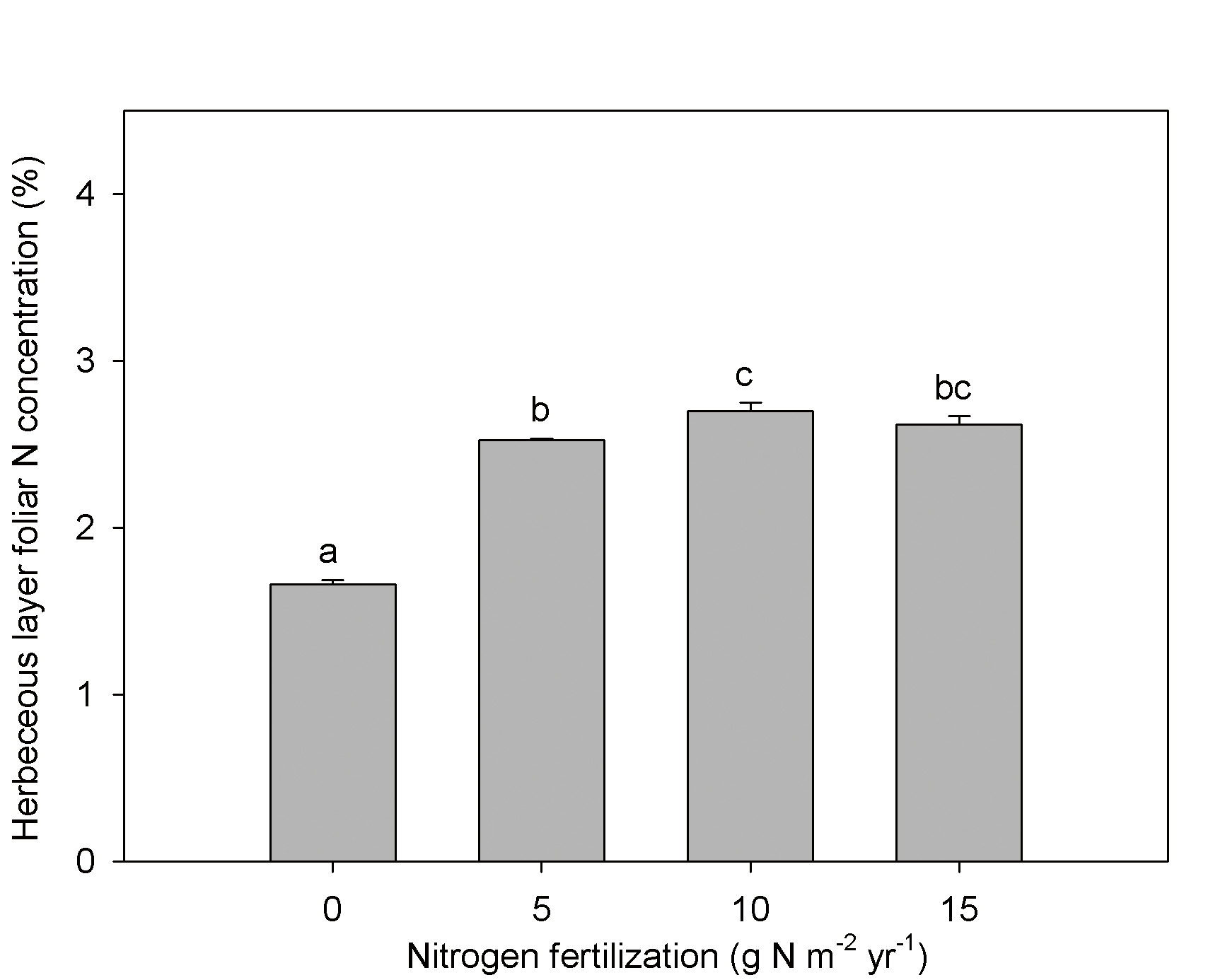

Supplement: Figure S3 — Effects of nitrogen (N) addition on foliar N concentrations of herbaceous layer plants. Significant differences among N treatments are indicated by different letters. (TIF) [file pone.0087975.s003.tif]

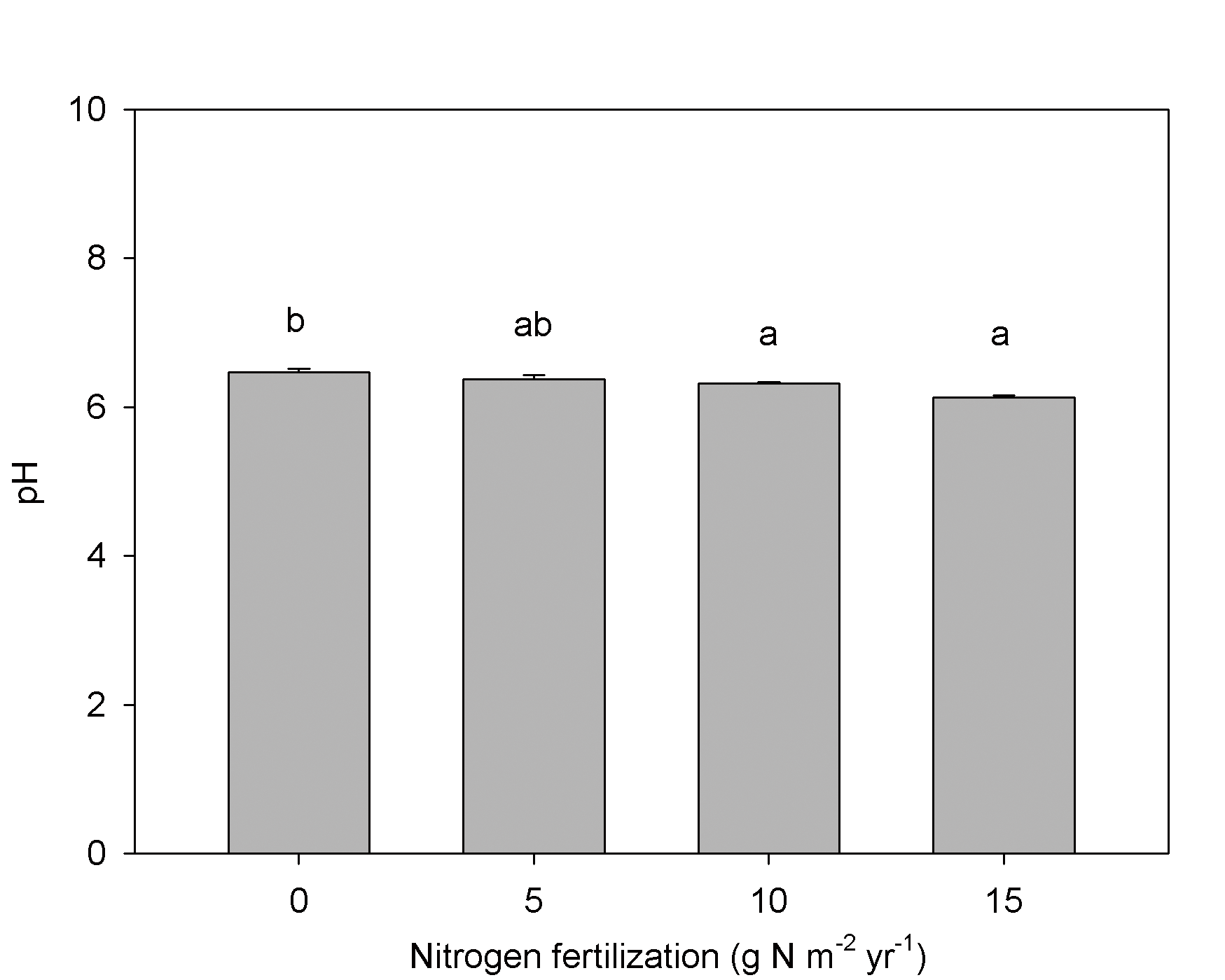

Supplement: Figure S4 — Soil pH in control and nitrogen (N) treatments plots after 3 years fertilization. Significant differences among N treatments are indicated by different letters. (TIF) [file pone.0087975.s004.tif]
